# Supplementary material for: SHIP1 deficiency causes inflammation-dependent retardation in skeletal growth
Source: Life Sci Alliance. 2024 Feb 22;7(5):e202302297. doi: 10.26508/lsa.202302297 (PMC10883774; doi:10.26508/lsa.202302297)
Supplement: Supplementary file 1 [file LSA-2023-02297_TableS1.docx]

#### Supplementary Table 1

Expression of the osteoclast marker genes in femoral diaphysis of *SHIP1^styx/styx^* versus WT mice*.*

| **Gene** | **log2FoldChange** | **Padj** |
| --- | --- | --- |
| *Traf6* | 0.070417 | 0.70194 |
| *Tnfrsf11a (RANK)** | 0.811596 | 0.000522 |
| *Ctsk* | 0.383539 | 0.363412 |
| *Itgav* | -0.4106 | 0.190816 |
| *Itgb3** | 0.665245 | 0.004044 |
| *Csf1r** | 0.487949 | 0.005803 |
| *Acp5* | 0.43014 | 0.34206 |
| *Nfatc1** | 0.341344 | 0.017942 |
| *Fos** | 0.725935 | 0.031977 |
| *Src* | 0.200533 | 0.385321 |
| *Clcn7** | 0.342409 | 0.004172 |
| *Atp6v1a** | 0.692628 | 6.15E-06 |
| *Dcstamp* | 0.71574 | 0.132148 |
| *Slc4a2* | 0.128695 | 0.526067 |
| *Tfrc* | -0.08671 | 0.796759 |
| *Slc11a2* | -0.26716 | 0.073806 |
| *Slc40a1** | -0.52676 | 0.000138 |
| *Ocstamp* | 0.2467 | 0.707038 |
| *Calcr** | 1.471911 | 0.002288 |
| *Mmp9** | 1.105579 | 3.25E-07 |
| *Car2* | -0.48572 | 0.084614 |

* Gene showing significant change in expression.
