# Supplementary material for: SHIP1 deficiency causes inflammation-dependent retardation in skeletal growth
Source: Life Sci Alliance. 2024 Feb 22;7(5):e202302297. doi: 10.26508/lsa.202302297 (PMC10883774; doi:10.26508/lsa.202302297)
Supplement: Supplementary file 2 [file LSA-2023-02297_TableS2.docx]

#### Supplementary Table 2

Expression of the osteoclast marker genes in femoral diaphysis of *Rag2^-/-^/Il2rg^-/-^/SHIP1^styx/styx^* versus *Rag2^-/-^/Il2rg^-/-^/SHIP1^+/+^* mice*.*

| **Gene** | **log2FoldChange** | **Padj** |
| --- | --- | --- |
| *Traf6* | 0.128484 | 0.642465 |
| *Tnfrsf11a (RANK)* | 0.550937 | 0.128001 |
| *Ctsk* | 0.404043 | 0.553651 |
| *Itgav* | -0.05508 | 0.948803 |
| *Itgb3* | 0.304214 | 0.463933 |
| *Csf1r* | 0.440549 | 0.094799 |
| *Acp5* | 0.617598 | 0.347949 |
| *Nfatc1* | 0.189634 | 0.454725 |
| *Fos* | 0.398554 | 0.510091 |
| *Src* | 0.161973 | 0.690725 |
| *Clcn7* | 0.203166 | 0.309074 |
| *Atp6v1a** | 0.454456 | 0.040189 |
| *Dcstamp* | 0.282163 | 0.771862 |
| *Slc4a2* | 0.134232 | 0.693467 |
| *Tfrc* | 0.039197 | 0.959233 |
| *Slc11a2* | -0.15558 | 0.560914 |
| *Slc40a1* | -0.38663 | 0.052927 |
| *Ocstamp* | 0.010595 | 1 |
| *Calcr* | 0.35237 | 0.736786 |
| *Mmp9* | 0.468837 | 0.190831 |
| *Car2* | -0.04619 | 0.955685 |

* Gene showing significant change in expression.
